# Modified Fixed Wall Oedometer When Considering Stress Dependence of Elastic Wave Velocities

**DOI:** 10.3390/s20216291

**Published:** 2020-11-05

**Authors:** Jong-Sub Lee, Geunwoo Park, Yong-Hoon Byun, Changho Lee

**Affiliations:** 1 School of Civil, Environmental and Architectural Engineering, Korea University, Seoul 02841, Korea; jongsub@korea.ac.kr (J.-S.L.); pytann@korea.ac.kr (G.P.); 2 School of Agricultural Civil & Bio-Industrial Engineering, Kyungpook National University, Daegu 41566, Korea; yhbyun@knu.ac.kr; 3 Department of Civil Engineering, Chonnam National University, Gwangju 61186, Korea

**Keywords:** compressibility, elastic wave, side friction, stress dependence

## Abstract

A modified oedometer cell for measuring the applied stresses and elastic waves at the top and bottom of the specimen is developed to evaluate the effect of the side friction on the stress dependence of the elastic wave velocities. In the modified cell, two load cells are installed at the top and bottom plates, respectively. To generate and detect the compressional and shear waves, a pair of piezo disk elements and a pair of bender elements are mounted at both the top and bottom plates. Experimental results show that the stresses measured at the bottom are smaller than those measured at the top during the loading and vice versa during unloading, regardless of the densities and heights of the specimens. Under nearly saturated conditions, the compressional wave velocities remain almost constant for the entire stress state. With plotting stresses measured at top, the shear wave velocities during unloading are greater than those during loading, whereas with plotting stresses measured at bottom, the shear wave velocities during unloading are smaller than those during loading owing to the side friction. The vertical effective stress may be simply determined from the average values of the stresses measured at the top and bottom of the specimens.

## 1. Introduction

The compression of soil is typically assumed to be one-dimensional when a vertical load is applied to the soil covering a large area. To simulate the one-dimensional condition, a one-dimensional compression test is widely conducted using an oedometric cell in the laboratory [1]. The time dependent stress–strain relationship under the zero-lateral strain condition (i.e., K_0_ condition) provides various deformation parameters of the soil.

Although many standards (e.g., ASTM D2435, BS 1377-part5, and DS/CEN ISO/TS 17892-5) suggest that the minimum ratio of the specimen diameter to height (D/H) should be 2.5 to minimize the side friction effect, specially designed large oedometers are often used for research purposes or for testing materials including large-sized granular materials, organic soils, municipal solid waste, mine tailings, and a mixture of soil and other materials [2,3,4,5,6,7,8]. When the elastic wave velocities are measured under one-dimensional conditions, relatively low ratio of D/H oedometric cells are applied to ensure the wave propagation length [9,10,11,12,13] because it is difficult to define the first arrival owing to the near field effect [14]. A low ratio of D/H increases the side friction, and the friction causes a lower change in the mean stress throughout the entire specimen than the change in stress at the top of the specimen.

In this study, series of experiments were conducted to explore the effect of the stress dependence on the elastic wave velocities owing to the side friction in a zero-lateral strain cell. An oedometric cell was modified to measure the applied effective stresses by installing load cells at the top and bottom of the specimen. Stress-dependent elastic wave characteristics and settlement were investigated by measuring the applied stresses at different locations.

## 2. Material and Methods

### 2.1. Test Materials

Silica sand (Kyungin Inc., Incheon, Korea) was used in this study. The specific gravity G_s_ for the sand (ASTM-D854) [15] was measured as 2.62. The extreme void ratios, e_max_ and e_min_, were obtained as e_max_ = 0.82 (ASTM-D4253) [16] and e_min_ = 0.56 (ASTM-D4254) [17]. The grain size distribution indicates that the tested sand is classified as poorly graded sand, SP based on the Unified Soil Classification System (ASTM-D421) [18] with a median grain size (D_50_) of 0.43 mm and a coefficient of uniformity (C_u_) of 1.35.

### 2.2. Zero-Lateral Strain Cell

**Figure 1:**
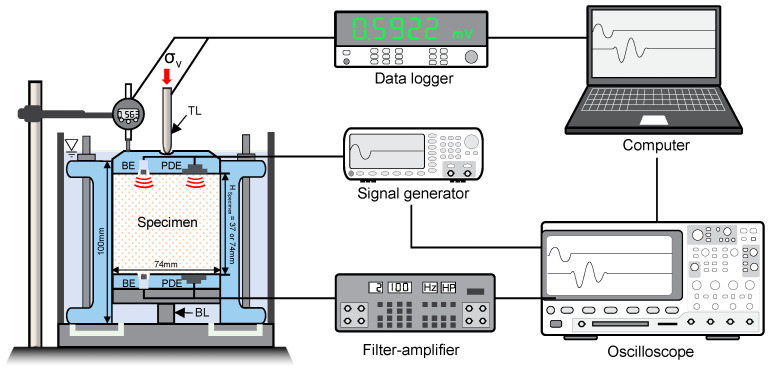
Schematic drawing of a modified oedometric cell with electronics. TL and BL denote the load cells installed at the top and bottom plates, respectively. In addition, BE and PDE denote the bender elements and piezo disk elements.

**Figure 2:**
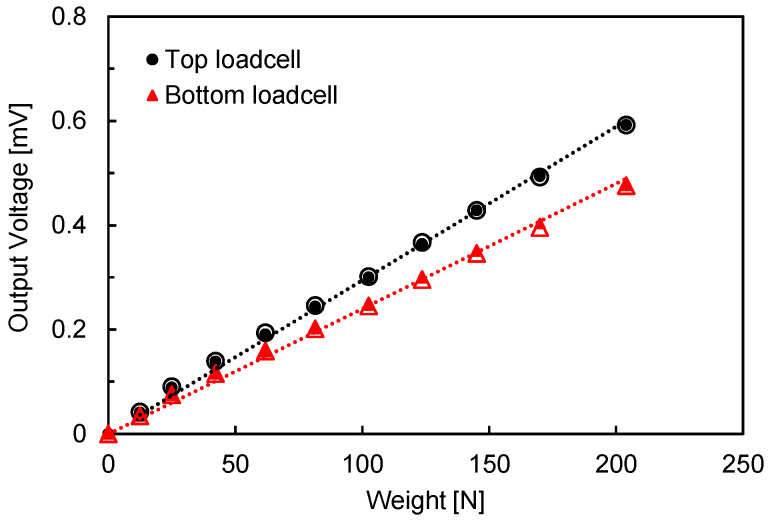
Calibration of load cells. Solid and hollow markers denote the loading and unloading steps, respectively.

A modified oedometric cell was used to measure the applied stresses at the top and bottom of the specimen while simultaneously measuring the compressional wave velocity (*V_P_*) and shear wave velocity (*V_S_*) under a zero-lateral strain condition, as shown in Figure 1. The cell has an inner diameter of 74 mm and a height of 100 mm. The electrical strain gauges (SG) with a 1.0 mm gauge length were attached to the load shaft to monitor the applied load at the top plate. A DC power supply (Agilent E3620A, Santa Clara, CA, USA) and data logger (Agilent 34972A, Santa Clara, CA, USA) were used to monitor the output voltage change of the strain gauge, which is amplified by a half-active Wheatstone bridge with two dummy gauges. Furthermore, to monitor the load at the bottom of the specimen, a load cell was installed beneath the center of the bottom plate. The gap between the wall and bottom plate was filled with vacuum grease to prevent soil from jamming within the gap. The two load cells were calibrated using the known applied stress, the results of which are shown in Figure 2. A thin layer of a lubricant was applied inside the oedometric cell to minimize the side friction between the specimen and cell.

The cell houses a pair of piezo disk elements (PDE) and a pair of bender elements (BE) at the top and bottom plates to generate and detect the compressional wave velocity *V_P_* and shear wave velocity *V_S_*, respectively. Peripheral electronics consist of a signal generator (Agilent 33500B, Santa Clara, CA, USA), a filter-amplifier (Krohn-Hite 3944), and an oscilloscope (Agilent DSO-X 3014A, Santa Clara, CA, USA) for elastic wave measurement. The elastic wave velocity can be calculated using the tip-to-tip distance between sensors *L_tip-to-tip_* and first arrival time *t_arrival_* [14]:

(1) $$V_{P}\;or\;V_{S}=\frac{L_{ti\mathrm{p-}t\mathrm{o-}tip}}{t_{arrival}}$$

### 2.3. Experimental Procedure

Sand specimens were prepared using a water-pluviation method and then vibrated to obtain predetermined density conditions (i.e., initial relative density Dr = ~40% and ~80%) to explore the effect of the specimen density with D/H = 1. Furthermore, two experimental cases were conducted for Dr = ~40% with D/H = 1 and 2 to assess the effect of the diameter-to-height ratio (D/H), as summarized in Table 1.

The applied vertical stress was doubled during each loading and unloading step. The maximum applied vertical stress was 640 kPa. The vertical settlements were continuously measured using an LVDT with a precision of 0.001 mm. The elastic wave velocities were measured at each step. Each loading step lasts until the vertical settlement converges to 0.001 mm/min.

## 3. Experimental Results and Discussions

### 3.1. Volume Change

**Figure 3:**
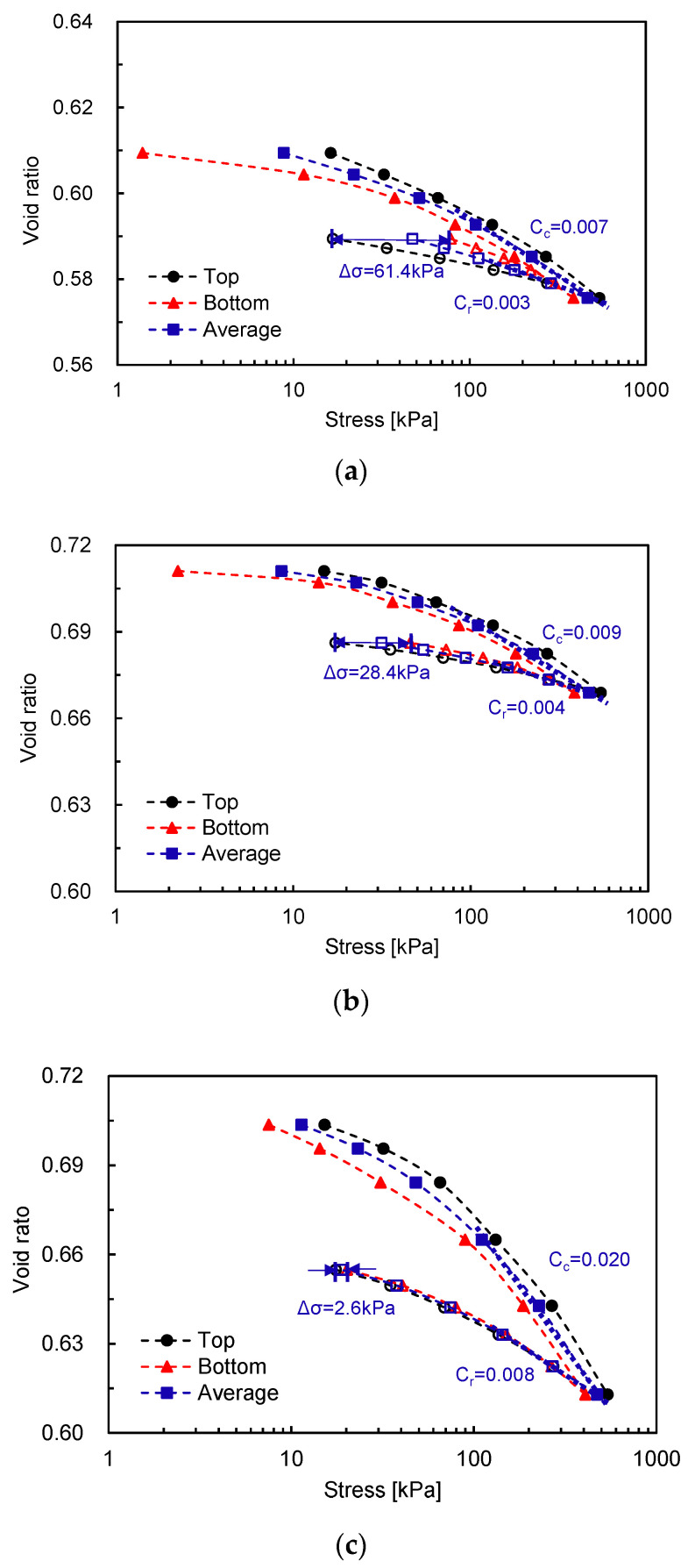
Variation in void ratio along the applied stress: (**a**) Dr = 80% and D/H = 1; (**b**) Dr = 40% and D/H = 1; and (**c**) Dr = 40% and D/H = 2. Note that Cc and Cr are calculated based on the average stresses. Solid and hollow markers denote the loading and unloading steps, respectively.

The measured void ratio–stress responses are plotted in Figure 3. As shown in Figure 3, similar trends are observed even for different densities and D/H. The void ratio gradually decreases with increasing applied vertical stresses. As shown in Figure 3, the measured stresses at the bottom show lower values than those measured at the top during the loading and vice versa during the unloading because the friction acts in the opposite direction of the applied load [19]. Previous studies reported that the high stress loss that occurs from friction is found either in the lower plasticity and/or over-consolidated clay (i.e., high stiffness specimen) because of higher interface friction [19,20]. Similarly, the specimen with a high Dr shows relatively high stress hysteresis at the given D/H ratio i.e., D/H = 1. The large stress variations measured at the top and bottom of a specimen with a low D/H ratio are also monitored. The compression index C_c_ and recompression index C_r_, which are calculated for the average stresses, indicate that a lower compressibility is observed: (1) for a higher density specimen under the same D/H; and (2) for a low D/H ratio specimen under the same Dr owing to side friction.

### 3.2. Elastic Wave Velocity

**Figure 4:**
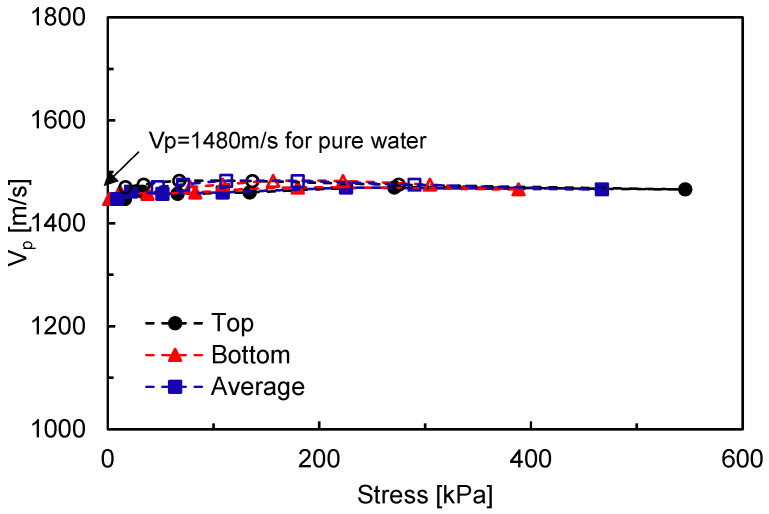
Evolution of compressional wave velocity along applied stresses for Dr = 80% and D/H = 1. Solid and hollow markers denote the loading and unloading steps, respectively.

At low frequencies, which are of engineering interest and where soil particles are assumed incompressible (i.e., in the case Gassmann’s equation is valid), *V_P_* can be expressed as a function of shear modulus *G*, bulk modulus of the solid skeleton *K_b_*, and total density *ρ* [21,22,23]:

(2) $$V_{P}={\left(\frac{4G/3+K_{b}/\left(1-B\right)}{\rho }\right)}^{1/2}$$

(3) $$\rho =\left(1-n\right)\rho_{s}+n\rho_{f}$$

where *n* is the porosity and *B* is the pore pressure coefficient. *ρ_s_* and *ρ_f_* indicate the densities of grain and fluid, respectively. Figure 4 shows the compressional wave velocity *V_P_* for the specimen of Dr = 80% and D/H = 1. As indicated in Figure 4, an almost constant value of *V_P_* (~1420 m/s) is observed with stress. Note that the compressional wave velocity of pure water is approximately 1480 m/s. Yang [22] proposed that the values of *B* and *V_P_* can be determined as a function of degree of saturation. When the Poisson’s ratio ν is assumed to be 0.3, the calculated value of *B* is 0.952 based on the measured *V_P_* = 1420 m/s, which indicates that the specimen was nearly saturated. In nearly saturated sand, the influence of the effective stress on *V_P_* is relatively small [24,25]. The experimental results also imply that the measured *V_P_* is rarely affected by a change in the effective stress in nearly saturated soils [22,26]. Note that similar results are observed for other experimental cases.

**Figure 5:**
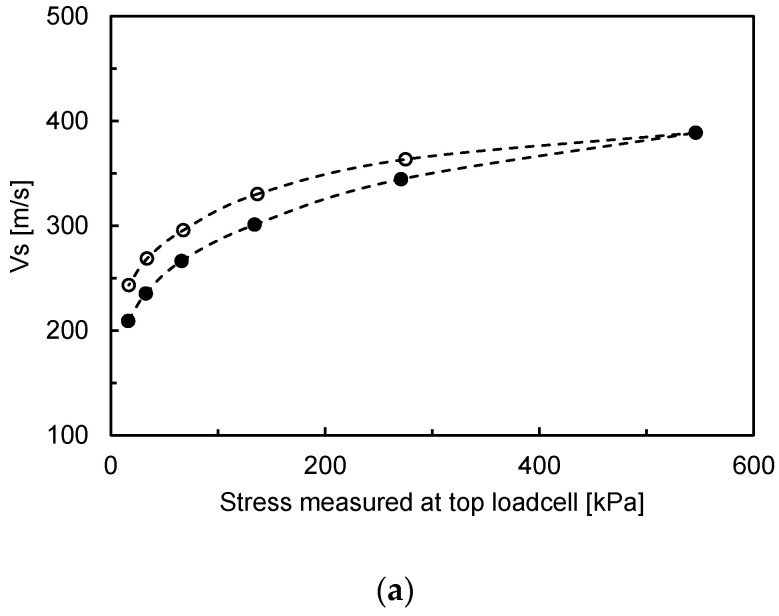

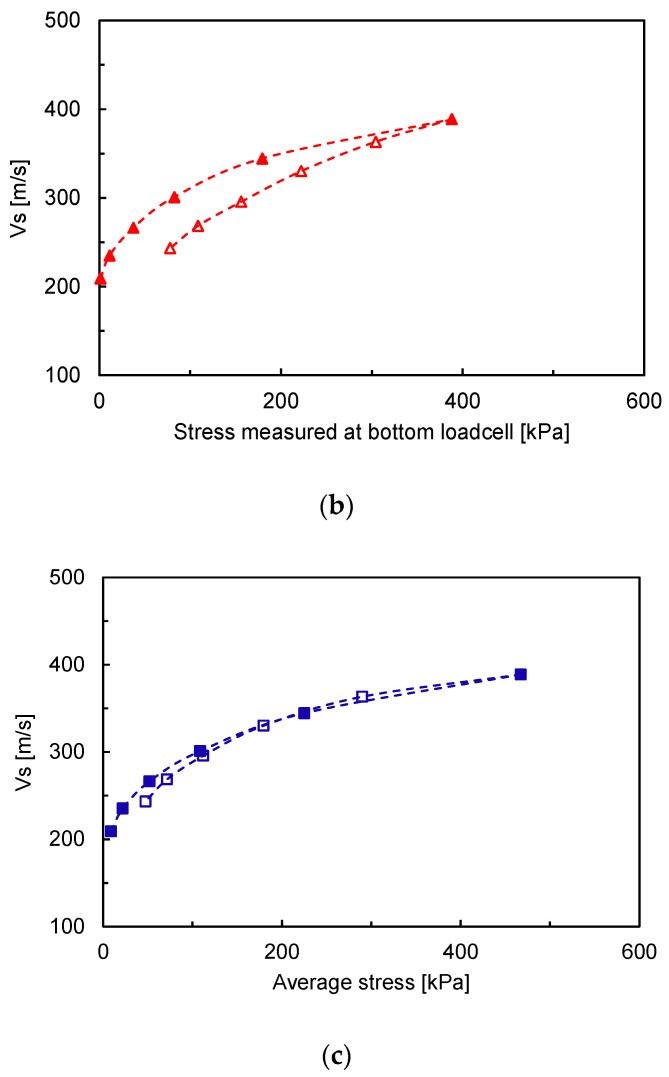
Variation in shear wave velocities for Dr = 80% and D/H = 1 along: (**a**) the applied stress at top; (**b**) applied stress at the bottom; and (**c**) the average applied stress. The solid and hollow markers denote the loading and unloading steps, respectively.

The velocity–stress relationship for the shear wave for any degree of saturation can be simply expressed as a power function of the mean effective stress (*σ*′*_m_*) in the direction of the wave propagation and in the direction of the particle motion [27,28,29] as follows:

(4) $$V_{S}=\alpha {\left(\frac{{\sigma^{\prime }}_{m}}{1\mathrm{kPa}}\right)}^{\beta }$$

where both *α* and *β* are experimentally determined. The *α* factor denotes the wave velocity at 1 kPa, and the *β* exponent indicates the stress sensitivity of the wave velocity. Note that Equation (4) is valid the stress-induced skeletal forces control the behavior of the media [30]. Figure 5 shows the measured shear wave velocity vs. for various stresses measured at different locations. The measured shear wave velocities for all specimens generally increase with increases in the vertical effective stress: With plotting the effective stress measured at the top, the values of vs. calculated during unloading are higher than those calculated during loading, whereas with plotting the effective stress monitored at the bottom, the values of vs. calculated during loading are higher than those calculated during unloading. When the measured vs. is plotted with the average vertical effective stress calculated from the top and bottom measurements, similar values of vs. are observed during the loading and unloading. Note that similar trends are observed for other specimens. When a fixed wall oedometric cell is used, the vertical effective stress applied at top of the specimen is not uniformly distributed but decreases with depth owing to the upward wall friction. Although the reduction in the actual effective stress applied in the whole specimen depends on the stress level, it was reported that the friction ratio of the wall friction to the applied stress is almost constant with the applied stress after a yield point (e.g., preconsolidation stress) [31,32]. Note that the friction ratio is approximately 10% of the applied stress [32]. As shown in Equation (4), the stress dependence of vs. can be defined with the mean normal effective stress on the polarization plane. To precisely establish the stress dependence of *V_S_*, the distribution of the vertical and horizontal effective stresses when considering the stress reduction should be known when a pair of bender elements is installed at the top and bottom of the specimen. The vertical effective stress within the specimen consists of the self-weight of the specimen and the applied stress at top. Lovisa and Sivakugan [33] reported that the applied stress governs the vertical effective stress within the specimen for small diameters such as cells used in a laboratory with maintaining D/H = 1, and, in general, it governs the vertical effective stress with the specimen when the applied stress is larger than approximately 20 kPa. Some researchers have suggested the theoretical distribution of the vertical effective stress corrected with the side friction of the cell [31,33]; however, the soil–wall resistance of the cell should be measured to determine the exact distribution of the vertical effective stress. Therefore, the vertical effective stress within the specimen when considering the side friction of the cell for a small oedometric cell can be simply and approximately determined based on the average values calculated based on the effective stresses measured at the top and bottom of the specimens. This is because both values indicate the maximum and minimum effective stresses of the specimen, respectively, assuming a linear distribution of the soil–cell interface resistance. Note that the horizontal effective stress can be calculated based on the coefficient of the earth pressure at rest (*K*_0_), which was determined from using the *K*_0_ equation as follows:

(5) $$K_{0}=1-\sin\Phi$$

where Φ is the internal friction angle of the soil. It is well known that the *K*_0_ equation proposed by Jaky [34] is commonly adopted to estimate the values of *K*_0_ in practice.

## 4. Conclusions

The modified zero-lateral strain cell was used to measure the stress-dependent geophysical properties and compressibility of the sand. The applied effective stresses were measured at the top and bottom of the specimen to investigate the side friction effect on the elastic wave velocity. The experimental results indicate that the measured compressional wave velocity (*V_P_*) is rarely affected by a change in the effective stress in the nearly saturated soils. When a pair of bender elements is installed at the top and bottom of the specimen, the stress reduction of the vertical effective stress by the side friction of the cell can be considered for precisely estimating the stress dependence of the shear wave velocity of the specimen. Although the distribution of soil–cell interface resistance is not linear along the specimen height, the vertical effective stress within the specimen for a small oedometric cell can be simply and approximately determined based on the average values calculated from the effective stresses measured at the top and bottom of the specimens.

## Figures and Tables

**Table 1:** Specimen conditions tested in this study.

No.	Relative Density (%)	Diameter-to-Height Ratio
1	80	1
2	40	1
3	40	2
